# Participant mothers’ attitudes toward genetic analysis in a birth cohort study

**DOI:** 10.1038/s10038-020-00894-7

**Published:** 2021-01-25

**Authors:** Midori Yamamoto, Kenichi Sakurai, Chisato Mori, Akira Hata

**Affiliations:** 1 Department of Sustainable Health Science, Center for Preventive Medical Sciences, Chiba University, 1-33 Yayoi-cho, Inage-ku, Chiba, 263-8522 Japan; 2 Department of Nutrition and Metabolic Medicine, Center for Preventive Medical Sciences, Chiba University, 1-33 Yayoi-cho, Inage-ku, Chiba, 263-8522 Japan; 3 Department of Bioenvironmental Medicine, Graduate School of Medicine, Chiba University, 1-8-1 Inohana, Chuo-ku, Chiba, 260-8670 Japan; 4 Chiba Foundation for Health Promotion and Disease Prevention, 32-14 Shinminato, Mihama-ku, Chiba, 261-0002 Japan

**Keywords:** Ethics, Genetics research

## Abstract

To conduct a long-term birth cohort study that includes genetic analysis, it is crucial to understand the attitudes of participants to genetic analysis and then take appropriate approaches for addressing their ambiguous and negative attitudes. This study aimed to explore participants’ attitudes toward genetic analysis and associated background factors among mothers who were enrolled in a large Japanese birth cohort. A questionnaire was sent to participants’ households, and the responses of 1762 mothers (34.0%) were used for the study. The majority of mothers recognized genetic analysis for themselves and their children and sharing of genetic data as beneficial. A low knowledge level of genomic terminology was associated with ambiguous attitudes toward genetic analysis and data sharing. Education level was positively associated with the recognition of the benefits of genetic analysis. Concern about handling genetic information was associated with the unacceptability of data sharing. Trust was associated with the approval of genetic analysis. Most mothers preferred that genetic analysis results be returned. These findings suggest the need for multiple efforts to maximize participants’ acceptance of genetic analysis, such as utilizing an educational approach to encourage familiarity with genetics/genomics, optimizing explanations for different educational levels, and explicitly disclosing the handling policy for genetic information.

## Introduction

A large number of birth cohort studies have been planned and conducted as important epidemiological research to understand the impact of environmental factors on children’s health and development [1, 2]. Some birth cohort studies include large-scale genetic analysis of children and their parents to explore gene–environment interactions in health and development [3, 4]. The Japan Environment and Children’s Study (JECS) has been conducted since 2010 as a large-scale nationwide birth cohort study of 100,000 children [5]. The JECS did not present any specific plan for genetic analysis at the initial informed consent process, but parents did provide consent for collecting biological samples, such as the mother’s blood, cord blood, and the father’s blood with the understanding that a portion of the donated blood would be stored for future genetic analysis [5, 6]. The samples and data collected are planned to be shared through the JECS biobank [5] and may be registered in access-controlled public databanks to contribute to further scientific research. Before genetic analysis and data sharing, the JECS will explain the research plan to participating parents and provide them opportunities to decline the use of their own and children’s samples for genetic analysis.

In birth cohort studies, which collect biological samples and data longitudinally, it is essential to gain participants’ understanding of the study and their trust in research institutions. Moreover, approval for genetic analysis must be obtained from as many participants as possible to explore gene–environment interactions. To maximize the number of participants who give their approval for genetic analysis and data sharing, it is crucial to understand the attitudes toward genetic analysis and related factors in mothers who are both participants and, at the same time, proxies for their children. Previous studies on the attitudes of public and research participants toward genomic cohort studies have reported that most people have positive feelings toward genetic testing, data sharing to a biorepository, and the return of individual genomic results, and attitudes toward genomics varied by education, nationality, gender, age, and marital status [7, 8]. Some Japanese studies reported that attitudes toward genetic analysis and research participation were associated with educational level and genomic literacy [9–13].

Mothers participating in birth cohort studies have commonalities in gender, age, having children, and donating biological samples, but they may have diverse attitudes toward genetic analysis. Moreover, studies involving children must address the ethical issue of protecting children’s privacy [14–16]. A systematic review of parents’ attitudes toward genetic testing reported that most parents viewed genetic testing on children as beneficial, and parents’ attitudes were associated with their education, knowledge of genetics, and children’s age [17]. As for parents’ attitudes toward birth cohort studies, some studies described the motivating factors and concerns for participation in the study [18–20]. However, little is known about the attitudes of mothers toward genetic analysis in a birth cohort study. In JECS, mothers are key proxies of children in most households and main contributors in follow-up studies. The present study aimed to clarify the attitudes of mothers participating in the JECS toward the benefits of genetic analysis and data sharing, and background factors related to their attitudes.

## Materials and methods

### Study population

Details of the JECS can be found in Kawamoto et al. [5]. A total of 97,415 pregnant women (103,062 pregnancies) were registered in the JECS at 15 regional centers located throughout Japan between January 2011 and March 2014. This study was conducted as an adjunct study of the JECS, whose research subjects were participants at the Chiba Regional Center. The study was designed with consideration, not to interfere with the JECS’ main study. A self-administered questionnaire was mailed, along with a regular newsletter, to 5176 households of mothers who were registered at the Chiba Regional Center as of December 2018 and was collected by mail. Since this study was a questionnaire survey, informed consent procedure was not performed, but the opportunity to refuse was ensured by stating in the questionnaire that the answers to the questionnaire were voluntary. This study was approved by the Research Ethics Committee of the Graduate School of Medicine, Chiba University.

### Data collection

The questionnaire included items for the following content areas: approving attitudes toward genetic analysis, concerns about handling genetic information, trust level in research institutions, preference for receiving results, self-rated knowledge level of genomic terminology, history of chronic disease or disability in the family, and the preference for receiving an explanation about genetic analysis. Data on the mothers’ age at delivery, educational background, and annual household income were collected from medical records or self-administered questionnaires completed during mid-to-late pregnancy [21]; the questionnaires were collected from the JECS dataset—“jecs-an-20180131”—released in March 2018.

Participants’ attitudes were assessed through several statements for which participants indicated their level of agreement. The items were as follows: for attitude toward genetic analysis, the item was “*To investigate the relationship between environment and children’s health, genetic analysis on children and parents is beneficial*,” for attitude toward data sharing, the item was “*If individuals are not identified, it is better to share the anonymous genetic information with other medical studies for the development of medicine*,” for concern about the security of genetic information, the item was “*I have a concern about the secure management of genetic information to avoid leakage*,” for concern about the secondary use of genetic information, the item was “*I have a concern that genetic information may be provided to other institutions without notice*,” for trust in research institutions, the item was “*The credibility of research institutions is one of my motivations for participating in the study*,” and for preference for receiving results, the item was “*I want to know the results of the genetic analysis*.” Answers were measured on a five-point Likert-type scale: “definitely agree,” “agree,” “unsure (not agree nor disagree),” “disagree,” and “definitely disagree.” Subsequently, “definitely agree” and “agree” were reclassified into “agree,” and “disagree” and “definitely disagree” were reclassified into “disagree.”

To examine participants’ basic knowledge of genetics, their self-rated knowledge level was assessed for the following five terms: “genome,” “gene,” “DNA,” “chromosome,” and “recombinant DNA technique,” following the procedure used in a previous study [10]. Participants were instructed to assign points to their knowledge level: 2 points for “knows the meaning of the term,” 1 point for “aware of the term,” or 0 points for “does not know the term.” When answers regarding the knowledge level were missing, they were given 0 points. The total score of the five items was grouped into tertile: low (0–6 points), medium (7–8 points), and high (9–10 points). The self-rated level of trust in the research institutions in cooperation with the JECS was measured on a five-point Likert-type scale. The level was classified with 1–2 points as “low,” 3 points as “medium,” and 4–5 points as “high.”

Participants’ preferences for receiving an explanation about the genetic analysis plan in the JECS was asked as follows: “We will explain the genetic analysis plan in the JECS by a written document. What other ways do you want to receive explanations?” The answer options were “no need except for documents,” “briefing session,” “individual face-to-face meeting,” “consulting corner in study-related events,” and “information service via telephone.” Multiple answers were allowed. The latter four answers were summarized as “verbal explanations” in the analysis.

### Statistical analysis

Multivariate multinomial logistic regression analyses were performed to identify factors associated with approving attitudes toward genetic analysis. The mother’s age, education, knowledge level of genomic terminology, trust in the research institutions, concern about the security of genetic information, and history of chronic disease in the family were included in the analysis models as independent variables. Concern about the secondary use of genetic information was not included in the analysis models because of its high correlation with concerns about security (Spearman’s correlation coefficient = 0.83). Structural equation modeling was used to explore the association of approving attitudes toward genetic analysis and related factors. A smaller Akaike’s information criterion (AIC) value was used to select the better-fitting model [22]. Model fit was assessed using the Goodness-of-Fit Index (GFI) ≥ 0.90, adjusted GFI (AGFI) ≥ 0.90, root mean square error of approximation ≤ 0.05, and comparative fit index (CFI) > 0.95. *P* < 0.05 was considered statistically significant. All analyses were conducted using SPSS ver. 25 (IBM Corp., Armonk, NY, USA).

## Results

### Respondent mother characteristics

Of the 5176 participants in the Chiba Regional Center, 1845 responded to the questionnaire (response rate 35.6%). After excluding the responses of non-mothers and mothers transferred from other regions and those with missing data for the variables used in the analysis, the remaining 1762 responses (34.0%) were used for the study. The children’s ages ranged from 4 to 7 years. The mothers’ ages ranged between 20 and 50 years, averaging 37.7 ± 4.8 years. Of the respondents, 67% were 30 years old or above, 69% had a college-level education (junior college or vocational and above), 67% were higher-income households (4 million yen per year and above), and 29% had family members with chronic diseases or disabilities (Table 1).

**Table 1:** Characteristics of mothers included in the study and all mothers at Chiba regional center of JECS

	Respondent mothers included in the analysis	All mothers participating in JECS in Chiba
	*N* (%)	*N* (%)
Number	1762	5176
Age at delivery in years		
<29	579 (32.9)	1930 (37.3)
30–34	629 (35.7)	1801 (34.8)
≥35	554 (31.4)	1444 (27.9)
Missing	0	1 (0.02)
Educational background		
Junior high	52 (3.0)	220 (4.3)
Senior high	494 (28.0)	1608 (31.1)
Junior college or vocational	777 (44.1)	2050 (39.6)
Undergraduate or above	439 (24.9)	1001 (19.3)
Missing	0	297 (5.7)
Annual household income during pregnancy (million Japanese Yen)		
<4	531 (30.1)	1674 (32.3)
4 to <6	610 (34.6)	1640 (31.7)
≥6	571 (32.4)	1399 (27.0)
Missing	50 (2.8)	463 (8.9)
History of chronic disease or disability in the family		
No	1247 (70.8)	NA
Yes	515 (29.2)	

*NA* not applicable

### Attitudes toward genetic analysis in the JECS and trust

Table 2 presents the mother’s attitudes toward genetic analysis and trust in research institutions. Of the 1762 responses, 74.3% agreed that “genetic analysis on children and parents is beneficial,” and 68.3% agreed that “it is better to share anonymous genetic information with other medical studies.” For these two items, 21.5% and 25.6% answered “unsure.”

**Table 2:** Maternal attitudes and concerns toward genetic analysis in JECS

	Response rate (%)		
	Agree	Unsure	Disagree
Benefit of genetic analysis			
Genetic analysis on children and parents is beneficial	74.3	21.5	4.1
Data sharing			
Anonymous genetic information is better to be shared with other medical studies	68.3	25.6	6.1
	Not concerned (disagree)	Unsure	Concerned (agree)
Concern about the security of genetic information			
I have a concern about the secure management of genetic information to avoid leakage	35.9	37.7	26.4
Concern about the secondary use of genetic information			
I have a concern that genetic information may be provided to other institutions without notice	33.4	35.9	30.8
	High (agree)	Medium	Low (disagree)
Trust level in research institutions			
The credibility of research institutions is one of my motivation to participation in the study	61.9	33.4	4.7
	Agree	Unsure	Disagree
Preference for receiving results			
I want to know the results of genetic analysis	89.3	8.7	2.0

Regarding concerns, 26.4% were concerned about the secure management of genetic information, and 30.8% were concerned about the secondary use of genetic information. A high level of trust in research institutions was reported by 61.9% of participants when expressed as the motivation to participate in the JECS. Most mothers (89.3%) expressed a preference to receive the results of genetic analysis. Although there were some differences in the ratio depending on the group of background factors, over 84% of all groups expressed a preference for the results (Supplementary Table 1).

### Self-rated knowledge level of genomic terminology

Of the five basic genomic terms, 29.0% answered that they did not know the term “genome.” Almost all mothers answered that they knew the meaning of or were aware of the term “gene,” “DNA,” and “chromosome,” but 0.2% to 0.6% did not know these terms (Table 3).

**Table 3:** Mothers’ self-rated knowledge of genomic terminology

	Know the meaning of the term (%)	Aware of the term (%)	Do not know the term (%)	No answer (%)
Genome	7.5	62.8	29.0	0.7
Gene	66.7	32.9	0.2	0.2
DNA	65.2	34.5	0.2	0.1
Chromosome	59.7	39.6	0.6	0.2
Recombinant DNA technique	34.9	61.9	2.9	0.3

### Relationship between attitudes toward genetic analysis and background factors

Table 4 shows the results of the multivariate multinomial logistic regression analysis for vague or negative attitudes toward genetic analysis. Mothers with higher knowledge levels of genomic terminology were less likely to answer that they were “unsure” about the benefit of genetic analysis and data sharing. Mothers with an educational level of junior high school had a higher tendency to disagree with the benefit of genetic analysis compared to those with other educational backgrounds. A high level of trust in the research institutions was associated with a low tendency to answer “unsure” or “disagree” regarding the benefit of genetic analysis and data sharing. Mothers who were concerned about the security of genetic information were likely to answer “unsure” to the benefit of genetic analysis and data sharing, and “disagree” to data sharing. Mothers whose families had a history of chronic disease or disability were less likely to disagree with genetic analysis and data sharing. When household income was included in the analysis models, the higher-income group (≥6 million yen) was less likely to answer “unsure” about the benefit of genetic analysis. The associations between approving attitudes and other factors remained after adjusting for household income (data not shown).

**Table 4:** Association between the non-approval attitudes toward genetic analysis and background factors

	*N*	Benefit of genetic analysis				Data sharing			
		Unsure		Disagree		Unsure		Disagree	
		aOR [95% CI]	*p* value	aOR [95% CI]	*p* value	aOR [95% CI]	*p* value	aOR [95% CI]	*p* value
Age at delivery in years									
<29	579	1.00 reference		1.00 reference		1.00 reference		1.00 reference	
30–34	629	0.86 [0.65, 1.16]	0.329	0.82 [0.46, 1.46]	0.491	0.90 [0.68, 1.20]	0.480	0.82 [0.50, 1.34]	0.424
≥35	554	1.00 [0.74, 1.34]	0.992	0.82 [0.45, 1.48]	0.501	1.06 [0.80, 1.41]	0.673	0.98 [0.60, 1.60]	0.928
Educational background									
Junior high	52	1.00		1.00		1.00		1.00	
Senior high	494	0.56 [0.29, 1.08]	0.082	0.23 [0.09, 0.60]	0.002	0.97 [0.51, 1.87]	0.935	1.50 [0.34, 6.62]	0.592
Junior college or vocational	777	0.48 [0.25, 0.93]	0.029	0.14 [0.05, 0.36]	<0.001	0.86 [0.45, 1.64]	0.648	1.31 [0.30, 5.73]	0.721
Undergraduate or above	439	0.36 [0.18, 0.73]	0.004	0.16 [0.06, 0.45]	0.001	0.76 [0.39, 1.51]	0.438	1.49 [0.33, 6.70]	0.605
Knowledge level of genomic terminology (score)									
Low (0–6)	663	1.00		1.00		1.00		1.00	
Medium (7–8)	636	0.92 [0.70, 1.20]	0.543	1.08 [0.61, 1.91]	0.797	0.84 [0.65, 1.09]	0.201	1.11 [0.67, 1.78]	0.661
High (8–10)	463	0.65 [0.47, 0.91]	0.011	1.31 [0.70, 2.45]	0.402	0.63 [0.47, 0.86]	0.004	0.95 [0.56, 1.62]	0.862
Trust level in the research institutions									
Low/medium	671	1.00		1.00		1.00		1.00	
High	1091	0.43 [0.34, 0.55]	<0.001	0.40 [0.25, 0.66]	<0.001	0.46 [0.36, 0.58]	<0.001	0.52 [0.34, 0.78]	0.002
Concern about the security of genetic information									
Concerned	465	1.54 [1.10, 2.15]	0.011	1.07 [0.58, 1.96]	0.830	2.11 [1.53, 2.91]	<0.001	2.16 [1.34, 3.51]	0.002
Unsure	664	2.22 [1.65, 2.98]	<0.001	0.92 [0.52, 1.65]	0.790	3.19 [2.40, 4.25]	<0.001	0.99 [0.58, 1.68]	0.963
Not concerned	633	1.00		1.00		1.00		1.00	
History of chronic disease or disability in the family									
No	1247	1.00		1.00		1.00		1.00	
Yes	515	0.72 [0.55, 0.94]	0.016	0.44 [0.24, 0.82]	0.009	0.85 [0.66, 1.09]	0.194	0.53 [0.32, 0.87]	0.013

Odds of attitudes “unsure” and “disagree” were compared to odds of “agree” by multinominal logistic regression analysis

*aOR* adjusted odds ratio, *CI* confidence interval

**Fig. 1:**
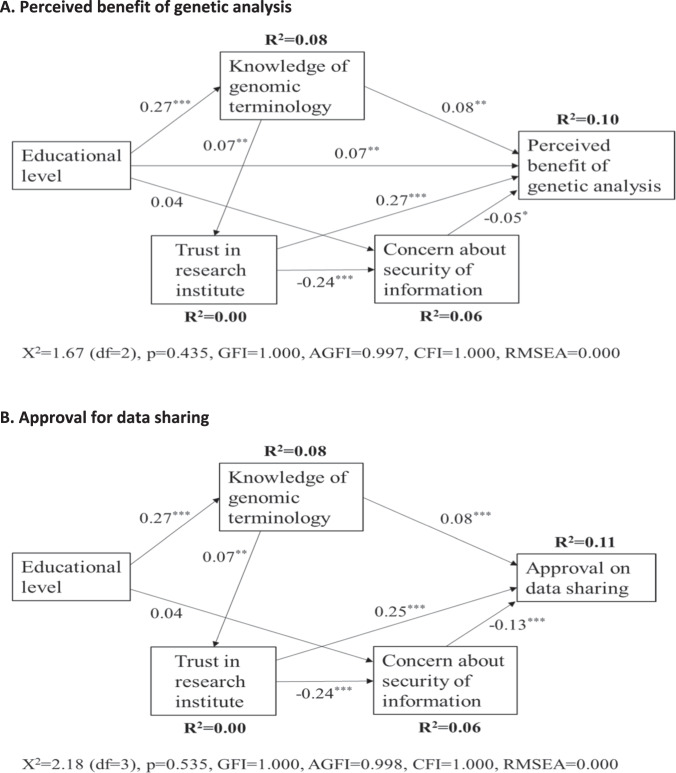
The path models for the relationship between approving attitude toward genetic analysis and background factors

Figure 1 shows the results of structural equation modeling for the association of approving attitudes toward genetic analysis and related factors. Of the five possible models for the benefit of genetic analysis (AIC = 27.7–36.4), the one with the lowest AIC was selected as the best model and was used for data sharing (AIC = 26.2). In both models, for the benefit of genetic analysis (A) and data sharing (B), the results of the chi-square test and other model fit indices showed that the models fit well. The level of trust and knowledge of genomic terminology was positively associated with approving attitudes toward the benefit of genetic analysis and data sharing, with trust being the most relevant (standardized estimates were 0.08 and 0.08 for the knowledge level, and 0.27 and 0.25 for trust, respectively). The negative association of concern about the security of genetic information was stronger with the attitudes toward data sharing than with the benefit of genetic analysis. Educational background was only associated with the benefit of genetic analysis. Among background factors, significant associations were found between education and the knowledge level of genomic terminology, knowledge level and trust, and trust and concern.

### Preference for explanation

**Fig. 2:**
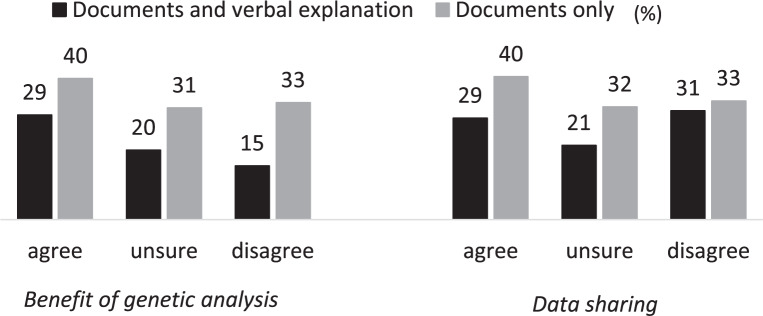
Preference for the method of explanation about genetic analysis. Data of those who answered “I don’t know” or did not answer about the method of explanation were not shown

As shown in Fig. 2, regardless of the attitudes toward genetic analysis and data sharing, 31–40% of mothers preferred no additional explanation other than written documents, and 15–31% preferred verbal explanations plus documents. Mothers who were uncertain or disagreed with the benefits of genetic analysis were less likely to express their desire for verbal explanations.

## Discussion

The present study clarified the attitudes and background factors among mothers of a birth cohort toward genetic analysis and data sharing. A mother’s recognition of the benefits of genetic analysis and data sharing was associated with trust in the research institutions, the knowledge level of genetic terminology, concern about the security of genetic information, and an educational background. However, there were some differences in the contribution of each factor to an approving attitude toward genetic analysis.

The majority of mothers recognized that the genetic analysis of children and parents is beneficial, and the benefits of genetic information are greater if they are shared with other medical studies. This finding was consistent with those from previous studies in Japan describing that the majority of participants favored genetic analysis [10–12]. Considering the low response rate of 34%, the responders may have been biased in favor of the study. Nevertheless, it is reasonable to assume that most mothers acknowledge the significance of the analyses in the JECS, as they have been involved in the JECS for more than 4 years and donated their biological samples. Meanwhile, 22–26% of mothers responded with “unsure” and 4–6% with “disagree.” Addressing vague or negative attitudes is pivotal to maximizing parents’ approval to use donated samples for genetic analysis. Therefore, we performed multivariate multiple logistic regression analysis to identify factors associated with ambiguous and negative attitudes. Additionally, we used structural equation modeling to investigate the associations between attitudes toward genetic analysis and related factors and the associations among the factors.

Trust in research institutions was consistently associated with approving attitudes toward the benefit of genetic analysis and data sharing. The results indicate that maintaining participants’ trust is critical for the acceptance of genetic analysis. In models adjusted for the effect of trust and other possible factors, a higher knowledge level of genomic terminology was associated with a decrease in “unsure” responses, but not with “disagree” responses. The knowledge level is considered to reflect a familiarity and interest in the field of genetics. As shown in the results, about 30–40% of mothers were unaware of the word “genome” and did not know the meaning of other basic terms, suggesting that many mothers had little knowledge of genetics. The “unsure” responses to the benefits of genetic analysis may be due to unfamiliarity with genetics or a lack of interest [23], in which case, approaches for improving participants’ familiarity with genetics/genomics may help them recognize the significance of genetic analysis. A study with the European public also reported that familiarity with genetics/genomics was a key difference between those who are “unsure” about donation and those who are “willing to donate” their data and DNA [24].

Previous studies reported that people with a higher educational background or genomic literacy were more likely to give favorable attitudes toward biorepositories and the use of anonymous data by other researchers [10, 12, 13, 25]. However, in this study, educational background was only associated with the recognition of the benefit of genetic analysis, but not with data sharing. Specifically, participants who indicated they “disagree” with the benefit of genetic analysis were more likely to be mothers with a low educational background of junior high school. This result suggests that the explanation of the significance of genetic analysis should be optimized for different educational levels. Designing explanation media that considers the comprehension level and interests of participants is required.

Mothers who were concerned about the security of genetic information management were more likely to disagree with data sharing with other medical studies. The concern was highly correlated with another concern about providing data to other institutions without notice to participants. Hence, the mothers may have a vague concern about the uncertainty regarding the handling of genetic information without distinguishing between information leakage, secondary use of information for non-research purposes, and access-controlled data sharing for research purposes. This finding is consistent with the research suggesting that many people who frequently express concern about data handling often seemed to confuse privacy, confidentiality, control, and security [26]. It is unclear whether mothers’ attitudes toward data sharing differ for their own data and their children’s data. However, concerns about confidentiality/privacy may be the reason for disagreeing with research using genetic information of themselves or their children [24, 27, 28]. An explicit explanation of the privacy policy for the genetic information of parents and children is essential to address various concerns. This explanation will enhance participants’ trust in research institutions.

As shown by the interrelationships among the attitudes toward data sharing, trust, and concern, the acceptance of data sharing seemed to depend on a balance between trust and privacy concerns. However, in studies of the European public, people with low trust levels were not relieved of their concerns after being provided with the security policy of genetic information. Instead, they tended to be unwilling to donate data and to have a concern about the use or misuse of DNA data by police and governments. [24, 29]. It is uncertain if this will be the case in mothers of birth cohort studies. Further studies are required to determine whether certain concerns lead to the refusal to allow genetic analysis. In any case, to enhance trust between participants and researchers, it is necessary to provide transparent information and interactive communication. Additionally, our study showed that trust was positively related to the knowledge level of genomic terminology. Efforts to improve familiarity with genetics/genomics may also enhance trust.

Most mothers preferred to be informed of the results of genetic analysis, although higher rates were found in mothers with high trust and without familial history of chronic disease. This study did not specify the type of genetic information, whose genetic information was being obtained or shared, and the reason they wished to know the genetic results. In public and biobank participants in Japan, many people have wished to learn about their genetic susceptibility to diseases [9, 30]. Mothers may have a similar interest in the genetic information for themselves and their children [31, 32]. Returning results would be a potent incentive for the public to participate in genetic research and might improve participants’ trust [8, 25, 33]. However, in addition to limited research budgets and manpower, returning results of genetic information may cause confusion with clinical results or misunderstandings [8], and additional ethical issues exist regarding parental access to children’s genetic information [15, 34]. In explaining the policy of returning results of genetic analysis, researchers should be prepared to address the mother’s considerable interest in genetic information.

The findings of our study suggest the importance of utilizing approaches to enhance familiarity with genetics/genomics and address various privacy concerns to improve mothers’ acceptance of genetic analysis. Educational activities such as workshops may be effective in increasing participants’ understanding [35]. However, face-to-face re-contact with participants is quite difficult in a longitudinal large-scale cohort study. In this study, many mothers preferred only written documents about genetic analysis, and those who disagreed with the benefit of genetic analysis tended not to prefer verbal explanations. Science communication activity may mainly reach those who are already interested in science, and people with low education may be less interested in science communication [36, 37]. Therefore, flexible approaches will be necessary, such as providing various explanatory media for participants with diverse educational levels as well as varying familiarity and interest in genetics/genomics. Further assessment of the effectiveness of these approaches will be needed to examine how the acceptance of genetic analysis and the related factors will be changed by the approaches.

The strength of this study was its focus on the negative or ambiguous attitudes toward genetic analysis in participant mothers of the birth cohort and to unveil the associated background factors. However, this study also has several limitations. First, attitudes and background factors other than basic characteristics were examined cross-sectionally, and the causal relationships could not be clarified. Second, the response rate for this survey was 35.6%. One reason for the low response rate may be that, unlike the JECS, there was no incentive payment for answering the questionnaire in this survey. Therefore, responses may have been biased toward those who were relatively supportive of the study. Among mothers in this study, the response rate was higher in those with a higher age and educational background. Given the relationship between educational background and attitudes, the proportion of respondents with favorable attitudes may be slightly higher than that of all mothers in the JECS. Third, the self-rated knowledge level of terminology may differ from actual understanding. Although more questions are needed to accurately measure participants’ genomic literacy, we used limited questions to avoid overburdening the participants. We believe that the self-rated knowledge level of terminology can be a predictor of familiarity with genetics/genomics. Finally, as this study was conducted for mothers registered in one regional center out of 15 centers, caution in generalizing the participants’ attitudes is needed. In addition, this study targeted mothers as proxies of children and main contributors to JECS. However, since about half of the children’s fathers are also JECS participants, it would be meaningful to investigate fathers’ attitudes toward genetic analysis in the future.

## Conclusion

This study found the following associations: (1) ambiguous attitudes toward genetic analysis among mothers with low familiarity with genetics/genomics, (2) denial of the benefit of genetic analysis among mothers with low educational backgrounds, (3) unacceptance of data sharing if mothers expressed concern about the handling of genetic information, and (4) approval of genetic analysis/data sharing if mothers had a high level of trust in research institutions. In addition to maintaining participants’ trust, approaches for enhancing familiarity with genetics/genomics, optimizing explanations for different educational levels, and explicitly disclosing privacy policies of genetic information will be needed to maximize participants’ acceptance of genetic analysis in birth cohort studies.

## Supplementary information

Supplementary Table 1
